# 

**DOI:** 10.1192/bjb.2017.27

**Published:** 2018-08

**Authors:** Lena Jawad

**Affiliations:** ST5 in General Psychiatry, Birmingham and Solihull Mental Health NHS Foundation Trust, London, UK; email: lenajawad@doctors.org.uk

*Still Down* by Dean F. MacKinnon, a US-based psychiatrist, is a patient-centred guide written primarily for patients – and their families – seeking effective management for treatment-resistant depression.

The book begins with a revision of the symptoms of major depressive disorder and a summary of how treatment has developed to include a wide range of antidepressant medications that allow patients to be trialled on alternative treatments when any one agent is unsuccessful. The author explains treatment-resistant depression – where patients fail to respond to antidepressant therapy – using nine case studies that suggest reasons for treatment failures, starting with relatively straightforward cases and ending with more complex scenarios. Scenarios include patients who have been inadequately treated or misdiagnosed as well as patients who are ‘treatment resistant.’ Strategies, both biological and psychological, based on the author's own clinical experiences are suggested as ways to overcome antidepressant failure. These are summarised in a table towards the end of the publication.

MacKinnon presents cases concisely, in an engaging and conversational style. Each presentation ends with a summary of the key (general) clinical points and case notes which highlight diagnostic features specific to the patient's individual presentation.

The main strength of this work is its clarity of information. Easy-to-read prose, bullet-points and tables help break up the text in ways that aid comprehension. Explanation of medical jargon where used and the relative absence of jargon ensures suitability to the target audience. Limitations include its focus on treatments approved by the U.S. Food and Drug Administration, which may be less relevant in countries other than the USA. Furthermore, the book does not include novel evidence on pharmacological or psychological treatments. Irrespective of this, it remains a valuable commodity for healthcare professionals, offering a general revision of the topic and inspiring an individualised approach to managing patients.

In summary, this is a well-written and helpful resource for patients and relatives seeking to gain a better understanding of depression and its management.

